# Editorial note

**DOI:** 10.1098/rstb.2021.0422

**Published:** 2022-01-03

This issue is the second part of a two-part issue on ‘Voice modulation: from origin and mechanism to social impact’. Part I can be found at https://royalsocietypublishing.org/toc/rstb/2021/376/1840. The Introduction to Part I contains an overview of both issues [1].
